# Assessment of the Porous Structure and Surface Chemistry of Activated Biocarbons Used for Methylene Blue Adsorption

**DOI:** 10.3390/molecules28134922

**Published:** 2023-06-22

**Authors:** Barbara Charmas, Magdalena Zięzio, Katarzyna Jedynak

**Affiliations:** 1 Institute of Chemical Sciences, Faculty of Chemistry, Maria Curie-Sklodowska University, Maria Curie-Sklodowska Sq. 3, 20-031 Lublin, Poland; 2 Institute of Chemistry, Jan Kochanowski University, Uniwersytecka Str. 7, 25-406 Kielce, Poland; kjedynak@ujk.edu.pl

**Keywords:** activated carbons, physical activation, microwave modification, porous structure characterization, surface chemistry, organic dye adsorption

## Abstract

In the presented research, activated carbons from wheat bran were obtained as a result of pyrolysis and physical activation (CO_2_ or/and steam). In addition, the obtained materials were subjected to additional modification with superheated steam using the microwave radiation as an energy source. The detailed materials characterization was performed using low-temperature nitrogen adsorption/desorption, Raman spectroscopy, X-ray diffraction, thermal analysis (TG), Boehm’s titration, point of zero charge (pH_pzc_), scanning electron microscopy (SEM) and FT-IR/ATR methods. Moreover, the sorption capacity towards methylene blue (MB) was determined. The activated carbons were characterized with a well-developed surface and pore structure (S_BET_ = 339.6–594.0 m^2^/g; V_p_ = 0.157–0.356 cm^3^/g). Activation in the presence of steam and additional modification with microwave radiation resulted in much better development of the porous structure (S_BET_ = 600.4 m^2^/g; V_p_ = 0.380 cm^3^/g). The materials were shown to possess amorphous structure and thermal stability up to the temperatures of ~450–500 °C. They have good adsorption capacity towards MB varying from 150 mg/g to 241 mg/g depending on activation manner. The adsorption can be described by the pseudo-second order model (R^2^ = 0.99) and fitted to the Langmuir isotherm.

## 1. Introduction

As it is commonly known, activated carbons can be produced from many materials containing carbon including organic waste materials. Currently, mainly household and agricultural wastes, as well as by-products of the agri-food industry, are used. These solid residues are applied owing to low cost, availability and large amounts [1]. For instance, peach kernels [2], olive kernels [3], almond shells [4], coffee grounds [5], walnut shells [6] or wheat bran [7] are commonly used for activated carbons preparation.

The selection of an appropriate starting material is of significant importance since the obtained carbon materials properties, i.e., porous structure or number and type of surface oxygen groups depend on the type and characteristics of the applied precursor. However, it is known that the activated carbon porosity depends not only on the raw material but also on the activation method and the pyrolysis process parameters [1].

Activation is a key step in the development of the pore structures of the carbon materials obtained by the carbonization process. This can be conducted in two ways, by physical or/and chemical activation [1,8]. Physical activation, also known as thermal activation, takes place in two stages [9]. The first stage includes the organic raw material carbonization (pyrolysis) and takes place in an inert atmosphere at temperatures in the range of 300–800 °C. In this stage, less stable bonds are broken, the volatile fraction (gases and tar) are released from the precursor and the so-called original porous structure is created. During the second stage, the previously obtained biochar is activated at temperatures 700–1000 °C in the presence of an activating agent. Partial gasification of the char takes place which results in the development of a porous structure and, thus, in an increase in its specific surface area. In the initial activation process phase, tarry products are eliminated, the previously formed pores are expanded and new ones are created. However, as the activation time increases, the specific surface area and pore volume decrease. In addition, the too long activation time favors the widening of the pores but not the deepening or the formation of new ones. As a result, mainly meso- and macroporous materials are obtained [10,11].

As the most commonly used physical oxidizing agents, one can distinguish: steam, carbon dioxide, air or mixtures of these gases [9]. The reaction with air is exothermic so it is difficult to maintain an appropriate, constant temperature. Additionally, oxygen is an aggressive reagent and its use as an oxidizing agent causes many difficulties. In many processes, activation with oxygen is conducted at low temperatures and is combined with the additional steam treatment. The course of the reactions is presented below [12,13]:

C + O_2_ → CO_2_    ΔH = −387 kJ/mol (1)

2C + O_2_ → 2CO     ΔH = −226 kJ/mol (2)

Steam and carbon dioxide are much more often used for activation. The reactions using steam or carbon dioxide require a temperature range of 700–950 °C and are endothermic, which facilitates the control of the process to a large extent [1]. The use of carbon dioxide is preferred due to its low reactivity at a high temperature which makes it easier to control the activation process. Moreover, the activation of CO_2_ promotes the formation of micropores while the activation of steam causes their expansion. Hence, the activated carbons pyrolyzed in the presence of steam are characterized by a smaller micropores volume and a larger volume of meso- and macropores [10,14]. The reaction course with the use of CO_2_ and steam is presented below [1,12,13,15]):

CO_2_:

C + CO_2_ → 2CO    ΔH = +159 kJ/mol (3)

steam:

C + H_2_O → H_2_ + CO    ΔH = +130 kJ/mol (4)

CO + H_2_O → CO_2_ + H_2_    ΔH = −42 kJ/mol (5)

However, carbon dioxide is characterized by a larger molecule size compared to steam, which results in slower diffusion of molecules in the channels of activated carbon pores, difficult access to micropores and a much slower reaction progress [12].

One of the effective methods for improving the carbon material’s porous structure is hydrothermal modification using the microwave as an energy source (MW). The microwave treatment is characterized by such exceptional properties as heating which is selective, fast and occurs in full volume; there is no contact between the heating device and the heated sample. Moreover, owing to the microwave modification, one can improve activated carbon quality and additional pollutants or hazardous substances are not produced which is why this method is environmentally friendly. On the other hand, there can occur some limitations such as undesirable interactions between microwave radiations and chemical substances [16,17].

The main aim of the paper was to prepare biocarbons from wheat bran by multi-stage pyrolysis in the CO_2_ atmosphere or modification using steam. The obtained activated carbons were subjected to hydrothermal activation with the superheated steam using microwave radiation as an energy source. First of all, the effects of different pyrolysis procedures and the additional microwave modification on the development of the porous structure, surface chemistry and the sorption properties of biocarbons were analyzed. Moreover, in these investigations, wheat bran was used as a precursor for obtaining biocarbons. Currently, there are not many literature reports on the use of wheat bran for the preparation of biocarbons which are used in the adsorption process of organic dyes.

## 2. Results and Discussion

### 2.1. Proximate Analysis

Table 1 presents the results of the proximate analysis performed for the starting material and the obtained activated biocarbons. The precursor contains 72.4% of volatile matter and only 18.3% of fixed carbon. These results are consistent with the results obtained by Lazdovica et al. [18] (76.3% of volatile matter and 18% of solid residues—char, coke).

The data presented in Table 1 show that the steam activation causes a significant reduction in the content of volatile matter (VM%), which indicates the ordering of carbon in biochars by incorporating it into the created organic structures. The reduction in VM% content is more intense during the modifications involving the microwave radiation (AC-1_MW_, AC-1-OX_MW_), but the smallest content of VM% is found in the biochars obtained in accordance with procedure 2. After introducing the 2 h isothermal stage at 400 °C, biochars with the smallest content of VC% (the difference in volatile matter between the samples AC-1 and AC-2 is 12.5%) and the largest content of FC% (AC-2; 51.7%) were obtained. The FC% content in all biochars increases compared to that of the starting material. The ashes content in the tested materials ranges from 16.9 to 35.2%. 

### 2.2. Textural Characteristics

Table 2 presents the data concerning the burning degree (%B) determined directly after the pyrolysis process, informing about the pyrolysis efficiency as well as the degree of surface development of the biochars. Analyzing the values, it can be observed that the atmosphere (CO_2_), the additional isothermal stage at 400 °C and the additional oxidizing agent (steam) intensively increase the burning degree and, thus, the surface development. For the materials pyrolyzed in the CO_2_ atmosphere, these values are in the range of 74–75%. The largest values of the %B were observed for the AC-1-OX sample (%B~85%) for which steam was an additional activating agent. Such a high %B indicates a significant loss of carbon and, at the same time, suggests large porosity of the obtained activated carbon. The difference in the burning degree for the material pyrolyzed in the CO_2_ atmosphere (AC-1) and in the presence of steam (AC-1-OX) is ~10% which indicates better expanding the porosity of activated biocarbon. Similar relationships for the other carbon materials were described in [1,8]. According to Wigmans [12], steam is a much more effective activating agent than CO_2_. The smaller molecular size of the steam allows faster diffusion through the pore channels as well as easier access to the micropores.

**Figure 1:**
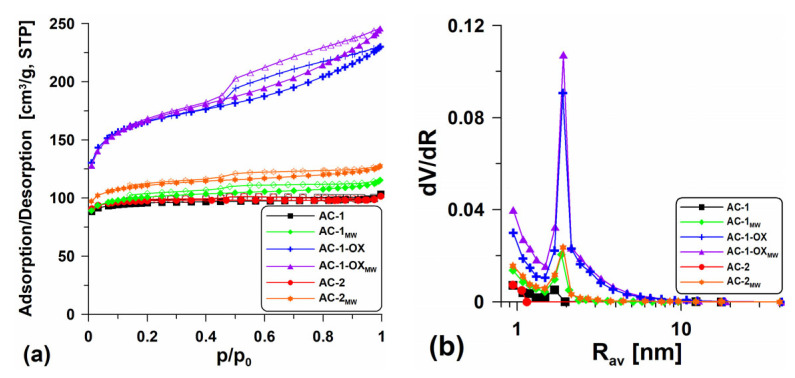
(**a**) Low-temperature nitrogen adsorption/desorption isotherms and (**b**) pore volume distribution curves in relation to their mean radii dV/dR = f(R_av_) for the obtained materials.

Table 2 presents the textural characteristics for all obtained activated carbons while Figure 1 shows the low-temperature nitrogen adsorption/desorption isotherms (Figure 1a) and the pore volume distribution curves in relation to their mean radii (Figure 1b). The shape of the isotherms determined for the samples AC-1 and AC-2 are type I isotherms without visible hysteresis loops (Figure 1a), characteristic of the microporous materials in which the monolayer adsorption occurs. Practically the isotherms overlap, suggesting only slight differences in the structural parameters (Table 2). Extending the intermediate stage (from 1 to 2 h) at 400 °C made the surface of the AC-2 material slightly better developed (S_BET_ = 346.7 m^2^/g, V_p_ = 0.179 cm^3^/g) than the surface of the sample obtained according to procedure 1 (AC-1; S_BET_ = 339.6 m^2^/g, V_p_ = 0.160 cm^3^/g). However, from an economic point of view, this procedure is unfavorable. The S_micro_ values are high and indicate the large microporosity of the materials. The shape of the pore size distribution curves versus the mean radii (Figure 1b) is characteristic of the materials with the homogeneous pore distribution with the dominant pore size R_dom_ ~2 nm.

It was very advantageous to introduce steam in the annealing stage at 800 °C (sample: AC-1-OX). The additional steam oxidation resulted in better development of the porous structure. The isotherm determined for this material is type IV (Figure 1a), characteristic of the mesoporous materials. The clearly visible H2-type hysteresis loop indicates the presence of bottle-shaped pores. The obtained material (AC-1-OX) has a well-developed specific surface area S_BET_ = 594.0 m^2^/g and a pore volume V_p_ = 0.356 cm^3^/g. On the pore volume distribution curves (Figure 1b), a peak indicating the presence of pores with R_dom_ ~2 nm and a certain share of micropores can be observed. 

In order to develop the biocarbons porosity, the additional steam modification using the microwave as an energy source was applied. This modification resulted in a slight increase in the specific surface area and better development of the porous structure of the materials (Table 2). On the isotherms (type I, Figure 1a), there appear poorly formed hysteresis loops, proving that the materials are microporous and contain a very small amount of mesopores. In the case of the AC-1-OX sample, the additional application of microwave radiation did not bring the expected results. The difference in the area between the AC-1-OX and AC-1-OX_MW_ samples is only 6 m^2^/g (Table 2). The shape of the isotherms obtained for AC-1-OX_MW_ indicates that they are type IV isotherms with the relatively well-developed H2-type hysteresis loop which indicates the presence of bottle-shaped mesopores. The mean pore radius (R_av_) values determined for the obtained materials are in the range of 0.9–1.3 nm.

For the tested materials, the volume of sorption pores (V_total_) and macropores (V_macro_) was also determined using methanol as an adsorbate (Table 2). These values are important because the obtained materials can be used as effective adsorbents for removing contaminations from the aquatic environment and macropores play an important role as the “transport channels”. One can see (Table 2) that the obtained values of V_total_ and V_macro_ are over 10 times larger than the volume of sorption pores. The activated carbon obtained in the oxidizing atmosphere of steam (V_total_ = 3.013 cm^3^/g) and that was additionally modified using the microwave as an energy source (V_total_ = 2.34 cm^3^/g) were characterized by a very large total volume of pores. Unfortunately, the additional use of microwave treatment resulted in the reduction in the sorption pores volume in the other discussed materials (V_total_~0.947–1.031 cm^3^/g). Significant V_total_ values resulted from the fact that methanol fills both the available sorption pores and the intergranular spaces between the carbon matter particles.

### 2.3. XRD

**Figure 2:**
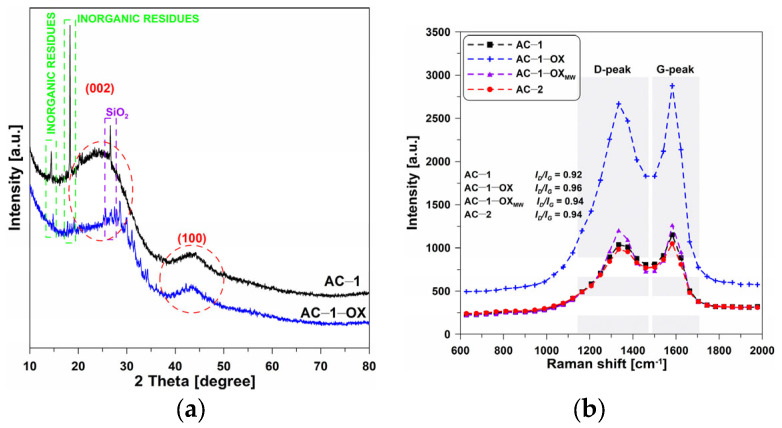
(**a**) XRD and (**b**) Raman spectra of the selected activated carbons.

Figure 2a presents the XRD spectra of selected activated carbons. One can observe the wide, bloated C(002) peaks, reaching the maximum at 2θ = ~23°, which are characteristic of the nongraphitic carbons structures. This indicates that the materials are mainly characterized by the amorphous structure [19].

Very low and wide C(100) diffraction peaks can be observed at 2θ = ~43° which indicates the presence of a small number of graphite structures. In addition, the presented diffractograms show very clear peaks at 2θ = 15° and 18° which can indicate the presence of inorganic residues from the wheat bran. Additionally, the peak at 2θ = 26.6° demonstrates the presence of SiO_2_ in ash [7]. The amorphous nature of the obtained materials was also confirmed by Raman spectroscopy.

### 2.4. Raman Spectroscopy

Figure 2b shows the Raman spectra obtained for the selected activated biocarbons. In the spectra, a significant increase in the intensity of the D and G bands for the AC-1-OX sample as compared to the other materials can be observed. On the other hand, no significant changes were found in the position of the G bands which determines the type of structure of the carbon material. For most tested activated carbons, the I_D_/I_G_ band ratio is 0.92–0.94. Such a result proves a small degree of graphitization of these materials and can also result from the poor ordering of the carbon material. 

The exception is the sample: AC-1-OX for which I_D_/I_G_ = 0.96, which indicates a slightly larger degree of graphitization [20,21,22] and better organization of the carbon deposit as a result of the use of steam as an additional oxidizing agent. The obtained results confirm the non-graphitic, i.e., amorphous, nature of activated carbons.

### 2.5. Thermal Analysis

**Figure 3:**
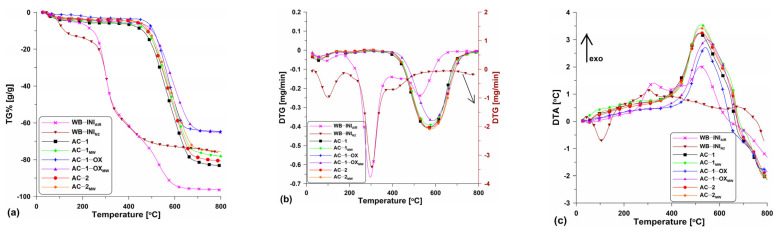
The course of the (**a**) TG%, (**b**) DTG and (**c**) DTA curves for the obtained activated carbons as well as for the initial material.

Figure 3 shows the TG (Figure 3a), DTG (Figure 3b) and DTA (Figure 3c) curves obtained for the tested materials. As the preliminary research, the thermal analysis of wheat bran in the air atmosphere was conducted. The research was aimed at the determination of the carbon content in the precursor. Based on the analysis of the curves (Figure 3a–c), it can be seen that the starting material contained approximately 5% of the moisture which was removed from the surface and pores to about 200 °C. The small moisture content demonstrates poor hygroscopicity and small porosity of the wheat bran. The main process of bran combustion was carried out in two stages. The first stage, up to the temperature of ~390 °C was related to the elimination of volatile organic compounds, the content of which was ~55% (Figure 3a). The second stage was a bit slower and was related to the degradation of the organic matter present in the sample. This stage is over at the temperature of ~650 °C with a mass loss of about 97%. Accompanying these two mass loss steps are the energy effects seen on the DTA curve (Figure 3c). Evaporation of moisture is the endothermic process while carbon oxidation is the exothermic one leading to the heat release. The analysis results showed that wheat bran, due to the large content of organic compounds, is a suitable precursor for the activated biocarbons.

As follows from the analysis in the N_2_ atmosphere, the thermal decomposition of wheat bran (WB-INI_N2_; Figure 3a–c) took place in three major steps: (I) in the temperature range 20 °C–200 °C and was related to the water evaporation, (II) in the temperature range of ~200 °C–440 °C the main loss mass step took place and it was due to the thermal degradation of organic compounds such as cellulose, starch, lignin, hemicellulose and protein components contained in the wheat bran. A series of reactions, for instance, decarboxylation, dehydration, deamination and fragmentation, took place; (III) the third step was related to the final mass loss in the temperature range of ~440 °C–800 °C. This was due to the pyrolysis of protein, lignin and residues charring [18].

The curves course shows that the obtained biocarbons are stable up to about 450–500 °C. However, the stability depends insignificantly on the isothermal stage time (400 °C), the atmosphere (CO_2_ or steam) and the energy source. In the case of unmodified samples (Figure 3a), a greater mass loss can be observed (~83% at 800 °C). In turn, the samples after the microwave modification are characterized by a slightly smaller material loss (at 800 °C it is ~75%). This means that these samples contain less carbon material as some may have been oxidized by the steam generated by means of microwave radiation.

The discussed differences can be also observed on the DTG (Figure 3b) and DTA (Figure 3c) curves. Clear peaks directed downwards are found on the DTG curves confirming the mass loss within a certain temperature range. These peaks are regular and wide which proves the equal speed and temperature range of the combustion process. The DTA curves (Figure 3c) show the energy effects of the processes which took place. With the temperature increase, clear peaks on the curves related to the exothermic process of activated carbons combustion can be observed. As follows from the course of the curves, the peak maxima are evidently “shifted” to the left pointing out that the combustion process for the individual samples started at different times.

### 2.6. SEM/EDX

**Figure 4:**
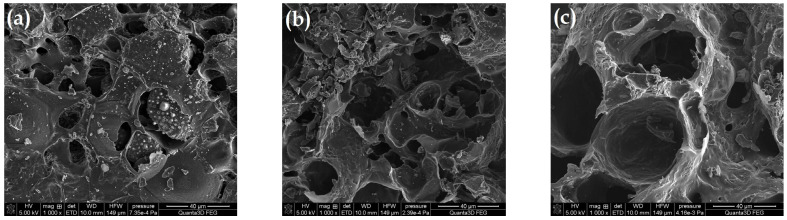
SEM images for (**a**) AC-1, (**b**) AC-1-OX and (**c**) AC-1-OX_MW_ samples (magnification ×1000).

Figure 4 presents the SEM images for the selected samples. Figure 4a presents the structure of the material pyrolyzed only in the CO_2_ presence. It is characterized by the heterogeneous structure and the smallest burning degree (%B; Table 1). The biocarbon surface contains numerous gaps and cavities of various sizes and shapes. Moreover, it is rough and folded. Pyrolysis in the oxidizing steam atmosphere caused the additional burning of the carbon material, making the pores accessible and open (Figure 4b). The use of modifications in the form of superheated steam assisted by the energy of microwave radiation resulted in the widening of the existing pores, indicating even better ordering of the carbon structure. The oxidizing atmosphere caused the disordered particles of the carbonaceous material to burn, which made the structure more homogeneous (Figure 4c). This material was characterized by the best-developed surface and porous structure (Table 1).

Table 3 presents the results of the EDX chemical composition microanalysis for the selected activated biocarbons. As one can see, the composition is characteristic of the materials obtained from organic wastes. The materials contained mostly carbon (~73.03–90.49%), oxygen (~3.81–11.28%), nitrogen (~1.22–2.69%), potassium (~1.04–7.05%) and phosphorous (~0.94–5.13). A small amount of Si originated from SiO_2_ ash [7]. This compound was also visible in the XRD spectra (Figure 2a). The presence of the other elements is due to the composition of the waste precursor which contains minerals such as K, Na and Mg. 

### 2.7. Surface Chemical Nature

The Boehm titration is used to quantify the oxygen-containing surface groups. In this method, determination of oxygen groups is based on their neutralization with bases of increasing strength (different pK_a_ values). It is assumed that the acidic oxygen groups are neutralized by the bases: NaHCO_3_ (pK_a_ = 6.4) neutralizes the carboxyl groups (the strongest), Na_2_CO_3_ (pK_a_ = 10.3) reacts with the carboxyl and lactone groups, and NaOH (pK_a_ = 15.7) neutralizes the carboxyl, lactones and phenolic groups. In turn, NaOEt (pK_a_ = 20.6) reacts with the basic oxygen groups (carbonyl-containing groups: pyrones and quinones) [23]. However, nowadays HCl is increasingly used for the neutralization of basic groups [24]. 

The pH_pzc_ values provide the information about pH at which the number of negative and positive charges is equal. It means that if the solution pH is >pH_pzc_ then the surface is negatively charged while. When the solution pH is <pH_pzc_, the surface is positively charged. Table 4 presents the number of the groups which are present on the materials surfaces and pH_pzc_ values.

It can be concluded that the obtained materials are characterized by the presence of both basic and acidic groups on their surface. They are mostly acidic groups and for all materials the total number of these groups is ~1.9 mEq/g (Table 4). After the additional modification with the superheated steam using the microwave radiation as an energy source, their number does not change. In the case of all samples no carboxylic groups are identified. The number of basic groups varies and depends on the pyrolysis procedure. The AC-1 and AC-2 samples are characterized by a larger content of basic groups (Table 4) in comparison to the other materials. In this case, using CO_2_ as an activating agent promoted the formation of oxygen basic groups. The application of the additional activation with steam was not favorable for the formation of basic surface groups. The content of these groups in carbon AC-1-OX is ~50% smaller than that in the pyrolyzed material without simultaneous activation with steam (AC-1). As follows, the additional modification using microwave heating causes a significant reduction in the number of basic groups. The pH_pzc_ values are in the range of 7.5–8.4 (Table 4). In the case of MB adsorption, taking into account the adsorbent–adsorbate electrostatic interactions, the cationic dye is adsorbed more intensively by the activated carbons with the negatively charged surface.

### 2.8. FT-IR/ATR

**Figure 5:**
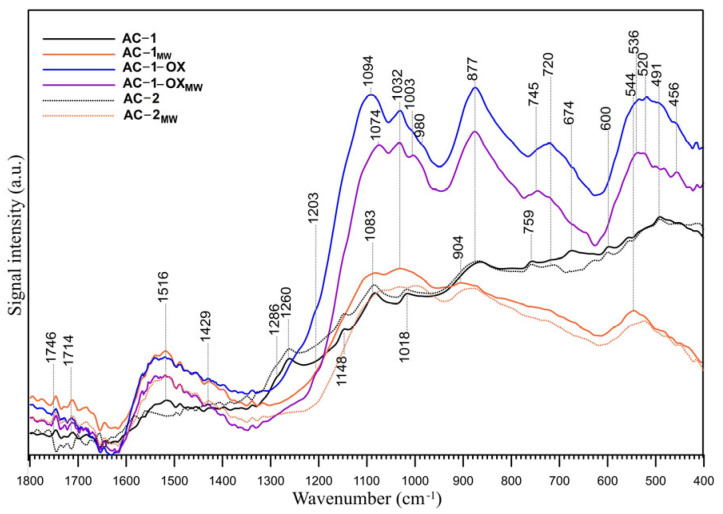
FT-IR/ATR spectra of the studied samples in the 1800–400 cm^−1^ range.

The FT-IR/ATR spectra of the samples are presented in Figure 5. The figure shows the spectra in the range of 1700–400 cm^−1^, which is the most suitable for the oxygen groups identification. The range of the C-H groups vibrations (4000–2000 cm^−1^) was neglected because no bands indicating the presence of these groups were observed in this range. Interpretation of the IR spectra of all carbonaceous materials is complicated because each of the functional groups visible in the IR spectrum may be responsible for the appearance of multiple bands in a wide range of wavenumbers and each band may have a contribution of many functional groups present on the surface of the sample [25]. The FT-IR/ATR spectra of the samples (Figure 5) are similar, and the difference resides mainly in the relative intensity of the peaks. The region below 1300 cm^− 1^ is particularly difficult to interpret in detail because many functional groups absorb the IR radiation within the same frequency range (C–O stretching modes in ethers, esters and alcohols, as well as C–OH bending modes in alcohols or amides). The bands below 1050 cm^−1^ can also have the contribution of inorganic compounds, i.e., silica and other inorganic compounds, caused by the presence of these compounds in the raw material. These modes are coupled with the other vibrations and do not exhibit well-defined group frequencies [26]. Nevertheless, some assignments are possible. Two peaks of very low intensity at 1746 and 1730 cm^−1^ are visible in all spectra, which may indicate the presence of a very small number of acidic carboxyl groups which have not been identified by the Boehm titration (Table 4).

The bands at ~1515 cm^−1^ are probably responsible for the carbon–carbon vibrations in the aromatic rings [27]. This can be confirmed by the presence of bands in the range of 900–650 cm^−1^, which are probably due to the C-H vibrations out of the plane of the aromatic ring [27]. The weak band (shoulder) observed at 1429 cm^−1^ is ascribed to the C-O-H stretching vibrations in phenols and has the smallest intensity in the AC-1 and AC-2 samples spectra. The spectra of the AC-1 and AC-2 samples display also bands at 1286 cm^−1^ (shoulder) and 1260 cm^−1^, which can be assigned to the lactone C-O-C structure [28]. Those bands are not visible in the spectra of the other samples. This observation seems to be confirmed by the Boehm titration (Table 4). However, those two peaks can be also compatible with the presence of the 4H-chromene, 2H-chromene and 2-pyrone basic groups. The bands at ~1203 cm^−1^ (shoulder) can originate from the phenolic hydroxyl group [29] and are visible in the AC-1-OX and AC-1-OX_MW_ samples spectra. The bands between 1200 and 950 cm^−1^ also indicate the presence of chromene and pyrone structures in the studied samples. Some of the bands in this range are also present in the spectra of samples subjected to oxidation (AC-1-OX) and modification by steam using microwaves as an energy source (AC-1-OX_MW_). In the case of oxidized samples, these bands are intense and shifted towards higher wavenumbers, which may indicate that they also contribute to the other oxygen functional groups. The overlapping peaks, which form the absorption bands in the 1300–1000 cm^−1^ region, can be also assigned to the ether (symmetrical stretching vibrations), epi-oxide and phenolic structures existing in the different structural environments [29]. The bands between 1230 and 1140 cm^−1^ are generally ascribed to the C-O stretching vibrations in the phenol and alcohol C-O groups. The bands at 1148, 1094, 1084, 600, 536 and 491 cm^−1^ could be due to C–O and C–O–C in the ether or alcohol/phenol structures and/or C=C stretching in the aromatic structures (C–C skeleton vibrations) [30]. The bands at ~720 cm^−1^ can result from the O-H out-of-plane bending in phenols. Those bands are the most intense in the AC-1-OX and AC-1-OX_MW_ samples spectra and a small peak is visible in the AC-2 spectrum. The results of the Boehm titration method, reported in Table 4, supported by the FT-IR spectra analysis indicate that the studied samples have an amphoteric acidic-basic character, with the presence of lactone acidic functional groups on the surface of AC-1 and AC-2 samples. However, there are some literature reports that suggest there is no clear correlation between the Boehm titration and the FT-IR data for quantifying carbon surface functionality [31].

### 2.9. Adsorption Kinetics

The presented materials were used in the adsorption process. In recent years, adsorption has become the most widely used method for water and wastewater treatment. This is a relatively cheap method and does not require expensive and complicated equipment [32,33]. Methylene blue was used as a model pollutant.

**Figure 6:**
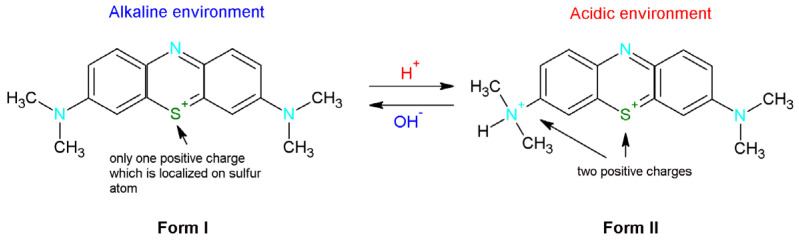
Methylene blue forms depending on the environment (alkaline or basic).

Methylene blue is classified as a basic thiazine dye. In the aqueous solution, this dye dissociates into a colored cation and a chloride anion [32]. Depending on the pH methylene blue can occur in two forms (Figure 6). In the basic environment MB is presented in the first form which means that there is only one positive charge which is localized on the sulfur atom. In turn, in the acidic solution one of the dimethylammonium groups is protonated [34]. Therefore, the new product is characterized by the presence of two positive charges.

In the case of the adsorption process, kinetics is an extremely important step that allows us to determine the substance absorption rate, e.g., from the liquid phase by the adsorbent particles. In other words, kinetics represents the adsorption efficiency of a given adsorbent, thus enabling the identification of its potential applications. However, it should be taken into account that adsorption is a complex process involving electrostatic, chemical and physical interactions. Thus, the rate of pollutants adsorption depends, inter alia, on the contact time of the adsorbent and the solution as well as on the diffusion process.

**Figure 7:**
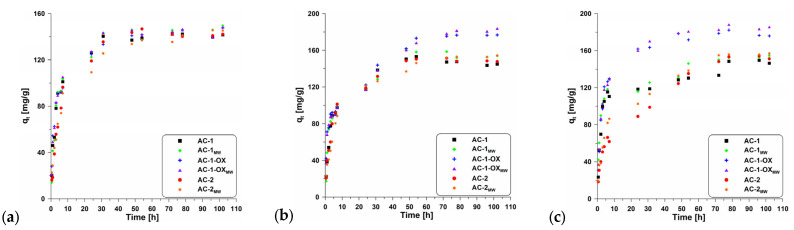
Adsorption kinetics of MB on the studied adsorbents at (**a**) 298 K, 303 K (**b**) and 308 K (**c**).

Figure 7 presents the effect of contact time on the adsorption of methylene blue onto the obtained activated carbons. The equilibrium state was achieved after about 50 h. According to the data (Table 5), the change in temperature did not significantly affect contact time; however, it can be observed that with increasing temperature, the sorption capacity increases.

The linear forms of pseudo-first order (PFO; Lagergren Equation; Equation (7)), pseudo-second order (PSO; Ho Equation; Equation (8)), Elovich kinetic (Equation (9)) and Weber–Morris intraparticle diffusion models (Equation (10)) were used to describe MB kinetics onto the tested adsorbents. The pseudo-first order kinetic model describes an adsorption rate that is directly proportional to the difference between the equilibrium and instantaneous adsorbate on the adsorbent surface. In turn, the pseudo-second order kinetic model assumes that the rate of occupancy of the accessible active sites by the adsorbate is proportional to the square of the number of vacant sites [35]. 

**Figure 8:**
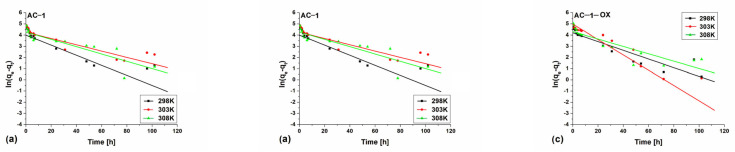
Pseudo-first order fitting model for the exemplary samples: (**a**) AC-1, (**b**) AC-1_MW_ and (**c**) AC-1-OX (symbols: ▪, ●, **Δ**—experimental points, lines—linear fittings, colors—different temperatures).

**Figure 9:**
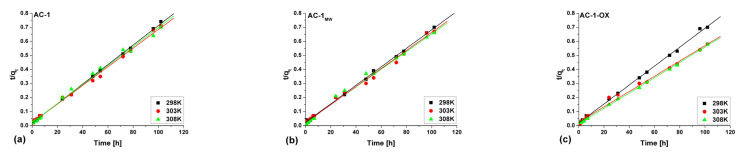
Pseudo-second order fitting model for the exemplary samples: (**a**) AC-1, (**b**) AC-1_MW_ and (**c**) AC-1-OX (symbols: ▪, ●, **Δ**—experimental points, lines—linear fittings, colors—different temperatures).

Figure 8 and Figure 9 show pseudo-first (Figure 8) and pseudo-second order fittings (Figure 9) for AC-1, AC-1_MW_ and AC-1-OX biocarbons. The data for AC-1-OX_MW_, AC-2 and AC-2_MW_ biochars are included in the SI (Figures S1 and S2). As follows from the data presented in Table 5 and in Figure 8 and Figure 9, Figures S1 and S2, taking into consideration the correlation coefficient R^2^ (R^2^ = 0.99), higher values are obtained by applying pseudo-second order equation. This means that the kinetics of the methylene blue adsorption process on the tested biochars are described by the PSO model. Fitting to this model shows that the rate of adsorption was largely dependent on the accessibility of active centers but not on the concentration of methylene blue in the solution.

In the case of the Elovich kinetic model, very large values of the correlation coefficient R^2^ = 0.96–0.98 (Table 5) were also obtained, meaning that the rate of adsorption was also influenced by the chemisorption process [35,36], which may occur on the surface and is characterized by the energetic heterogeneity.

In order to investigate the mechanism controlling the kinetics of the dye adsorption process on the obtained materials, the intraparticle diffusion model proposed by Weber–Morris [35] was used. Plotting the relationship q_t_ = f(t^1/2^), it is possible to determine the mechanism of the adsorption process in a given system. This dependence can be single or multi-line. In the case when the relationship is rectilinear over the entire range and passes through the origin of the coordinate system, intraparticle diffusion is the step controlling the adsorption process. On the other hand, when the rectilinear relationship does not pass through the origin of the coordinate system, it means that intra-particle diffusion is involved in the adsorption process, but this is not a speed-controlling step in the adsorption process. When the relationship is multi-linear, the intra-particle diffusion model shows that there are two or more stages that make up and influence the adsorption process: (a) the first (the fastest) stage is related to the external surface adsorption—the adsorbed molecules move from the solution to the surface of the adsorbent by diffusion through the boundary layer (diffusion in the boundary film); (b) the second stage includes the gradual diffusion of the adsorbate through the pores of the adsorbent (intra-particle diffusion); (c) the third stage is a state of equilibrium which includes very slow diffusion of the adsorbate from the larger pores to the smaller ones (micropores) [34,35,37,38,39].

**Figure 10:**
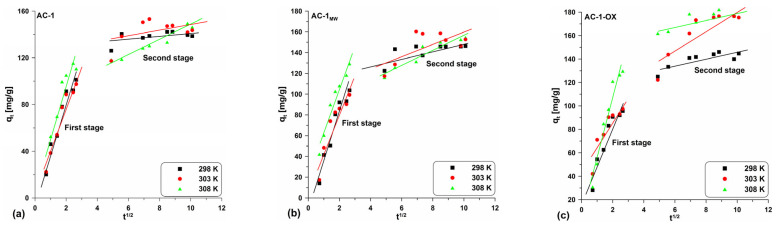
Intra-particle diffusion model for the exemplary samples: (**a**) AC-1, (**b**) AC-1_MW_ and (**c**) AC-1-OX (symbols: ▪, ●, **Δ**—experimental points, lines—linear fittings, colors—different temperatures).

Table 6 presents the parameter values determined from the equation linear form (Equation (9)) and Figure 10 and Figure S3 shows the curve courses. One can observe the presence of multi-line relationships indicating two stages (Figure 10 and Figure S3; Table 6). This demonstrates that not only intraparticle diffusion can have an influence on the adsorption rate, but also other processes in the system [34].

In this case, the first stage is related to the intraparticle diffusion and the second one is due to achieving the equilibrium by the system. Moreover, in all cases, the C parameters have positive values and they are different from zero. Based on these considerations, it can be concluded that intraparticle diffusion affects the adsorption rate, but this is not the stage that limits the whole process.

### 2.10. Adsorption Isotherm

In order to describe the manner of the adsorption process, two isotherm models were used. Figure S4 show experimental adsorption isotherms of MB on tested materials at temperatures of 298 K, 303 K and 308 K, whereas in Figure S5, the linear fitting according to Langmuir (Figure S5a–f) and Freundlich (Figure S5g–l) models are presented. Table 7 contains the calculated parameters (linear fitting) for the Langmuir and Freundlich models. As can be observed, with the increasing temperature, the values of q_e,exp_ increase. This means that adsorption can be described as an endothermic process [34]. All tested materials acquired the maximum adsorption capacity at 303 K, which indicates the influence of temperature on the adsorption process. These dependencies can result from the increase in dye molecule diffusion rate throughout the external boundary layer or greater mobility of some methylene blue molecules at higher temperatures which facilitates diffusion in the porous structure of activated carbons. 

The AC-1-OX_MW_ sample is characterized by the greatest adsorption capacity, which at the temperatures of 298 K, 303 K and 308 K is 212.59 mg/g, 220.79 mg/g and 241.95 mg/g, respectively. The AC-1-OX material (activated biocarbon) also exhibits good adsorption properties (Table 7); however, the q_e,exp_ values are smaller in comparison to the previously discussed sample. The AC-1, AC-1_MW_, AC-2 and AC-2_MW_ samples are characterized by much smaller sorption capacity (152–174 mg/g) compared to the materials activated in the steam atmosphere and additionally modified by the use of microwave energy. These differences can be due to two factors: porous structure and surface chemical nature. Taking into account the first factor, the materials which exhibit the best sorption properties (AC-1-OX, AC-1-OX_MW_, Table 7) are characterized by the well-developed surface (Table 2) as well as the micro/mesoporous structure (Figure 1a) while the other materials contain mainly micropores. According to the literature, the length of MB molecules is ~1.447 nm [40] which is why they diffuse faster and easier through the pores with a larger diameter such as mesopores. Moreover, the microwave treatment resulted in the extension of already existing pores which made it easier for the dye molecules to penetrate the pores interior and occupy active centers. 

The presence of oxygen-containing groups on the surface of the material also affected the adsorption process. The insignificant reduction in acidic groups after the microwave modification was favorable for the adsorption process since these kinds of groups can limit adsorption. The acidic groups can occupy some active centers which become inaccessible to the dye molecules—the interactions between the activated carbons surface and the MB molecules are reduced. Moreover, repulsive forces between the positively charged carbon surface and the dye molecules can also confine the whole process [41]. One can also observe that the basic groups also confined the adsorption because they could make active centers inaccessible. The materials which are characterized by a reduced amount of surface functional groups (after the microwave modification) exhibit a larger adsorption capacity. 

Table 7 shows the calculated values of the variables in the linear forms of the isotherm models used for the adsorption process modeling. As can be seen from the presented data, in the case of the Langmuir isotherm model very high values of the R^2^ correlation coefficient (R^2^ > 0.97) were obtained. The q_m,cal_ values differ from those obtained experimentally (q_e,exp_). These differences can result from the different influence of the surface functional groups that affected the adsorption process. In the case of the Freundlich model, the values of the R^2^ are slightly smaller in comparison to the Langmuir one. The K_F_ coefficient is high for all tested materials and describes the strength of the interactions between the adsorbate and the adsorbent. The n values are >2 and can indicate a significant contribution of chemical adsorption. In conclusion, in our case, the Langmuir model is suitable for the adsorption process description.

## 3. Materials and Methods

All reagents, methanol (Merck), hydrochloric acid (HCl—35%), sodium hydroxide (NaOH), sodium carbonate (Na_2_CO_3_) and sodium bicarbonate (NaHCO_3_) obtained from Standard (Poland), were analytical grade and commercially available. The methylene blue (M = 319.86 g/mol; λ_max_ = 663–667 nm) was obtained from Chempur (Poland). The wheat bran used as a precursor came directly from the grain milling in the household.

### 3.1. Material Preparation

Wheat bran was sieved and a fraction above 1 mm was selected for further research. The precursor was initially dried at 100 °C for 24 h. The pyrolysis was performed in the oxidizing atmosphere of CO_2_ (100 mL/min) according to the presented temperature program:

- temperature increasing up to 400 °C (10 °C/min);
- isothermal stage at 400 °C for 1 h (AC-1) or 2 h (AC-2);
- sample annealing at 800 °C for 3 h.

A multi-stage pyrolysis was applied introducing an isothermal stage at 400 °C. The main purpose of this treatment was to reduce the amount of formed by-products as well as to obtain materials with a well-developed porous structure. For the porosity development the steam activation was applied. The process was conducted as for the AC-1 sample, however, during the annealing stage (800 °C) steam was introduced into the system (0.6 mL/min). The sample was designated AC-1-OX.

The obtained activated biocarbons were subjected to the additional activation with the superheated steam using the microwave radiation as an energy source (60 min; 100%; 47–50 atm) (microwave reactor, Nano 2000; Plazmatronika, Wrocław, Poland). The redistilled water was used as the modifying agent. The obtained samples were designated by adding the “MW” to the symbol of activated carbon, e.g., AC-1_MW_. 

### 3.2. Characterization of Biochars

The porous structure of the biochars was determined using nitrogen adsorption–desorption isotherms at −196 °C (Micrometrics, ASAP 2405, Norcross, GA, USA). The standard parameters: specific surface area S_BET_ [42], pore volume V_p_ and pore size distribution dV/dR = (R_av_) [43] were determined on the base of experimental isotherms data. 

In order to assess the thermal stability, the thermogravimetric analysis was performed (Derivatograph-C, F. Paulik, L. Erdey, MOM, Budapest, Hungary). Al_2_O_3_ was used as the reference sample. The analyses were performed in the air or N_2_ atmospheres in the temperature range 20–1200 °C (10 °C/min). TG, DTG and DTA curves were recorded. Additionally, the preliminary thermal analysis was performed for the starting material in the nitrogen (designation: WB-INI_N2_) and the air (designation: WB-INI_AIR_) atmospheres. Thermals were also applied to perform the analysis of the content of volatile matter (VM), content of ash (A%) and the content of fixed carbon matter (FC%). 

The total pores (V_total_) and the macropores volumes (V_macro_) were determined by filling of the pores by methanol according to the manner described in [44].

For the determination of the amounts of acidic and basic oxygen functionalities, Boehm’s potentiometric titration method was used [45]. The pH_pzc_ (zero charge point) was characterized by the drift method [46]. 

A Meridian Diamond ATR Spectrometer (Harrick, Nicolet 6700) was used for infrared spectra recording. The 4000–400 cm^−1^ range spectra were obtained from the coaddition of 512 scans with 4 cm^−1^ resolution. All ATR spectra were corrected for water vapor and carbon dioxide. All spectral measurements were performed at least in triplicate.

The phase composition analysis was made with the powder diffractometer PANalytical Empyrean using the Cu-Kα radiation (40 kV, 25 mA, λ = 1.541874). The crystal structure analysis was made using the PDF-4+ 2019 Database.

The carbon matter structure was determined using the Raman spectra by means of InVia Renishaw Raman spectrometer (Raman Station 400 F, Perkin Elmer, UK) equipped with argon laser (514.5 nm). The spectra were recorded with the resolution of 1 cm^−1^. 

The samples morphology was analyzed using the DualBeam Quanta 3D FEG FEI microscope under the conditions of low vacuum at the accelerating voltage of 5 kV. The quantitative and qualitative analyses were performed using the energy-dispersive X-ray spectroscopy (SEM/EDX, acceleration: 15 kV).

### 3.3. Adsorption Kinetics

The batch method was applied for methylene blue adsorption. The research was carried out at 298 K, 303 K and 308 K. The methylene blue solutions (C_0_ = 600 mg/L; V = 0.025 L) were added to the flasks containing activated carbons (0.05 g). The flasks were placed on a shaker (84 rpm; 102 h). A sample was taken at appropriate intervals to evaluate the concentration of dye remaining in the solution. The measurements were made spectrophotometrically at the wavelength of 664 nm (“Helios Gamma”, Spectro-Lab, Lublin, Poland). The amount of adsorbed dye q_e_ at equilibrium was calculated from the following equation (Equation (6)):

q_e_ = ((C_0_ − C_e_) ∙ V)/m (6)

where: q_e_—the amount of adsorbed dye per 1 g of adsorbent at equilibrium [mg g^−1^]; C_0_—the initial concentration of the dye solution [mg L^−1^]; C_e_—the equilibrium concentration of the dye solution [mg L^−1^]; V—the dye solution volume [L]; m—the sample weight [g].

To describe the kinetic process, the linear form equations of PFO (Equation (7)), PSO (Equation (8)), Elovich kinetic model (Equation (9)) and intraparticle diffusion model (Equation (10)) were used [47,48]:

ln(q_e_ − q_t_) = lnq_e_ − k_1_∙t (7)

t/q_t_ = 1/(k_2_ ∙ q_e_^2^) + t/q_e_ (8)

q_t_ = 1/β ln (α ∙ β) + 1/β lnt (9)

q_t_ = k_d_ ∙ t^1/2^+ C (10)

where: q_t_—the amount of adsorbed substance per 1 g of adsorbent after time t [mg g^−1^]; q_e_—the amount of adsorbed dye per 1 g of adsorbent at equilibrium [mg g^−1^]; k_1_—the reaction rate constant [h^−1^]; k_2_—the reaction rate constant [g mg^−1^ h^−1^]; α—the initial adsorption rate [mg g^−1^ h^−1^]; β—the desorption constant [g mg^−1^]; k_d_—the intraparticle diffusion rate constant [mg g^−1^ h^1/2^]; C—the boundary layer thickness [mg g^−1^]; t—the adsorption time [h].

### 3.4. Determination of Adsorption Isotherm

The dye solutions with concentrations in the range of 50–1500 mg/L were used to determine the adsorption isotherms. Exact amounts of activated carbons (0.05 g) were placed in the flasks and the dye solutions (0.025 L) were added. The flasks were placed on a shaker (75 rpm; 50 h). The tests were carried out at three temperatures: 298 K, 303 K and 308 K. 

To describe the adsorption isotherms, the Langmuir (Equation (11)) and Freundlich (Equation (12)) models were used [49,50,51]:

C_e_/q_e_ = 1/q_m_ ∙ C_e_ + 1/q_m_ ∙ K_L_ (11)

logq_e_ = logK_F_ + 1/n logC_e_ (12)

where: q_e_—the amount of adsorbed substance per 1 g of adsorbent in the equilibrium state [mg g^−1^]; q_m_—the maximum amount of adsorbed substance [mg g^−1^]; C_e_—the equilibrium concentration of the dye solution [mg L^−1^]; K_L_—the Langmuir adsorption equilibrium constant [L mg^−1^]; K_F_—the Freundlich constant which indicates the adsorption capacity [mg^1–1/n^ (dm^3^)^1/n^ g^−1^]; n—the Freundlich adsorption intensity constant.

## 4. Conclusions

Wheat bran, which contains a large content of organic matter, is a suitable precursor for obtaining activated biocarbons. The obtained materials are characterized mainly by microporous nature; however, they contain also an insignificant amount of mesopores. The pyrolysis process carried out in the presence of steam caused porous structure development. The additional modification with the superheated steam using microwave radiation as an energy source resulted in the extension of the already existing pores, which resulted in a better surface development in the case of modified materials. The activated carbons show an amphoteric acidic-basic character, however, with a predominance of acid groups. On the basis of XRD and Raman spectra, it can be concluded that biochars are characterized by the amorphous structure. Generally, the materials are stable up to the temperatures 450–550 °C; however, their thermal stability depends on the pyrolysis and activation procedure. The biocarbons have a very good sorption capacity towards methylene blue (AC-1-OX_MW_ = 241.95 mg/g; 303 K). The adsorption process is described by the pseudo-second order model and fitted to the Langmuir isotherm (R^2^ = 99).

## Figures and Tables

**Table 1:** The volatile matter, ash and fixed carbon contents.

Sample	Volatile Matter [%]	Ash [%]	Fixed Carbon [%]
Wheat bran	72.4	8.7	18.3
AC-1	39.1	16.9	38.9
AC-1_MW_	28.5	22.4	44.8
AC-1-OX	31.4	35.2	31.8
AC-1-OX_MW_	19.8	36.3	40.3
AC-2	24.6	19.9	51.7
AC-2_MW_	19.9	26.0	50.8

**Table 2:** Textural characteristics of the obtained activated biocarbons.

Sample Designation	S_BET_	S_micro_	V_p_	V_micro_	V_total_	V_macro_	R_av_	%B
AC-1	339.6	307.7	0.160	0.134	1.252	1.092	0.94	75.4
AC-1_MW_	358.4	298.6	0.179	0.130	1.031	0.852	0.99	-
AC-1-OX	594.0	405.7	0.356	0.174	3.013	2.657	1.20	84.4
AC-1-OX_MW_	600.4	367.4	0.380	0.156	2.340	1.960	1.28	-
AC-2	346.7	320.5	0.157	0.140	1.686	1.529	0.91	74.6
AC-2_MW_	393.9	320.0	0.197	0.139	1.286	1.089	1.00	-

where: S_BET_—the specific surface area [m²/g]; S_micro_—the micropores surface [m²/g]; V_p_—the pore volume determined using the data from the N_2_ adsorption/desorption [cm^3^/g]; V_micro_—the micropores volume [cm^3^/g]; V_total_—the total pore volume determined using methanol [cm^3^/g]; V_macro_—the macropores volume, V_macro_ = V_total_₋V_p_ [cm^3^/g]; R_av_—the average pore radius [nm]; %B—the degree of burning.

**Table 3:** Results of the EDX chemical composition microanalysis.

Element/Line [%*w*/*w*]	Sample Designation			
	AC-1	AC-1-OX	AC-1-OX_MW_	AC-2
C/K	90.48	73.03	83.89	86.57
N/K	2.24	1.22	1.92	2.69
O/K	3.81	11.28	6.13	7.08
Mg/K	0.06	1.53	0.72	0.27
Si/K	-	-	-	0.18
P/K	0.94	5.13	2.41	1.18
S/K	0.06	-	0.05	0.08
K/K	1.04	7.05	3.98	1.70
Ca/K	1.17	0.52	0.60	-
Mn/K	0.19	0.16	0.16	0.21
Fe/K	-	0.09	0.14	-

**Table 4:** Surface functional groups present on the activated carbons surface and pH_pzc_ values.

Sample Designation	Content of Oxygen Surface Groups by Boehm’s Titration [mEq/g]				pH_pzc_
	Phenolic	Lactonic	Acidic Groups Total Number	Basic Groups Total Number	
AC-1	1.642	0.307	1.949	1.370	7.7
AC-1_MW_	1.936	-	1.936	0.511	7.5
AC-1-OX	1.883	-	1.883	0.696	8.4
AC-1-OX _MW_	1.820	-	1.820	0.233	7.6
AC-2	1.638	0.351	1.989	1.202	8.4
AC-2_MW_	1.872	-	1.872	0.808	7.6

**Table 5:** The calculated variables in the linear forms of the kinetic models.

	T [K]		PFO		PSO		Elovich Kinetic Model		
Sample Designation		q_e,exp_	k_1_	R^2^	k_2_	R^2^	α	β	R^2^
AC-1	298	141.7	4.21 × 10^−4^	0.85	2.25 × 10^−3^	0.99	6.66	4.5 × 10^−2^	0.95
	303	144.92	2.74 × 10^−4^	0.82	2.11 × 10^−3^	0.99	5.17	4.1 × 10^−2^	0.96
	308	148.96	3.13 × 10^−4^	0.77	1.80 × 10^−3^	0.99	15.83	5.2 × 10^−2^	0.88
AC-1_MW_	298	149.60	6.09 × 10^−4^	0.86	1.80 × 10^−3^	0.99	5.20	4.1 × 10^−2^	0.95
	303	154.04	3.04 × 10^−4^	0.67	1.89 × 10^−3^	0.99	5.72	4 × 10^−2^	0.95
	308	157.36	3.62 × 10^−4^	0.90	2.12 × 10^−3^	0.99	32.36	5.6 × 10^−2^	0.89
AC-1-OX	298	147.90	3.73 × 10^−4^	0.86	2.25 × 10^−3^	0.99	8.79	4.8 × 10^−2^	0.97
	303	176.80	6.67 × 10^−4^	0.83	1.14 × 10^−3^	0.99	7.36	3.9 × 10^−2^	0.96
	308	179.08	3.23 × 10^−4^	0.78	2.08 × 10^−3^	0.99	8.61	3.7 × 10^−2^	0.95
AC-1-OX_MW_	298	148.15	4.80 × 10^−4^	0.68	1.14 × 10^−3^	0.97	7.34	4.4 × 10^−2^	0.95
	303	183.63	5.88 × 10^−4^	0.95	9.62 × 10^−4^	0.99	6.09	3.7 × 10^−2^	0.96
	308	188.87	3.92 × 10^−4^	0.87	1.92 × 10^−3^	0.99	8.43	3.6 × 10^−2^	0.96
AC-2	298	142.20	3.14 × 10^−4^	0.76	1.29 × 10^−3^	0.99	2.61	3.7 × 10^−2^	0.96
	303	147.73	3.73 × 10^−4^	0.74	1.64 × 10^−3^	0.99	5.17	4.1 × 10^−2^	0.97
	308	153.93	4.11 × 10^−4^	0.89	7.06 × 10^−4^	0.97	2.13	3.7 × 10^−2^	0.95
AC-2_MW_	298	145.40	4.80 × 10^−4^	0.91	1.13 × 10^−3^	0.99	3.06	3.9 × 10^−2^	0.98
	303	154.42	4.71 × 10^−4^	0.97	2.12 × 10^−3^	0.99	2.76	3.6 × 10^−2^	0.98
	308	156.93	5.00 × 10^−4^	0.93	9.23 × 10^−4^	0.99	2.70	3.7 × 10^−2^	0.97

where: k_1_—the pseudo-first order reaction rate constant [h^−1^]; k_2_—the pseudo-second order reaction rate constant [g mg^−1^ h^−1^]; α—the initial adsorption rate [mg g^−1^ h^−1^]; β—the desorption constant [mg g^−1^].

**Table 6:** The calculated parameters values in the linear forms of the intra-particle diffusion model.

Sample Designation	T [K]	I Stage		
		k_d1_	C_1_	R^2^
AC-1	298	39.91	0.747	0.93
	303	39.02	0.361	0.93
	308	45.67	4.391	0.90
AC-1_MW_	298	44.22	7.553	0.93
	303	36.82	8.225	0.85
	308	42.63	20.17	0.95
AC-1-OX	298	32.83	16.35	0.89
	303	24.32	38.71	0.81
	308	52.16	2.508	0.94
AC-1-OX_MW_	298	39.15	4.836	0.92
	303	25.27	35.15	0.79
	308	48.19	10.64	0.94
AC-2	298	41.16	17.99	0.98
	303	40.13	4.874	0.98
	308	23.55	6.119	0.95
AC-2_MW_	298	33.80	3.943	0.98
	303	32.63	1.457	0.97
	308	34.27	3.681	0.98

where: k_d1_—the intra-particle diffusion rate constant of the first stage [mg g^−1^ h^−1/2^]; C_1_—the boundary layer thickness of the first stage [mg g^−1^].

**Table 7:** The calculated values of the variables in the linear forms of the isotherm models.

	T [K]		Langmuir			Freundlich		
Sample Designation		q_e,exp_	q_m,calc_	K_L_	R^2^	n	K_F_	R^2^
AC-1	298	152.71	199.70	0.0036	0.99	2.20	6.99	0.95
	303	154.18	197.29	0.0046	0.97	2.41	9.58	0.93
	308	159.13	197.56	0.0052	0.98	2.51	11.06	0.87
AC-1_MW_	298	159.49	193.74	0.0042	0.99	2.27	7.79	0.94
	303	168.71	192.78	0.0072	0.99	2.73	14.26	0.97
	308	174.96	188.78	0.0136	0.99	3.29	23.32	0.96
AC-1-OX	298	192.79	194.99	0.0111	0.98	3.37	26.60	0.96
	303	195.35	198.13	0.0164	0.99	3.78	36.50	0.96
	308	211.91	227.14	0.0212	0.99	4.08	45.29	0.95
AC-1-OX_MW_	298	212.59	225.31	0.0033	0.97	2.04	8.09	0.95
	303	220.79	231.54	0.0026	0.98	1.92	7.33	0.97
	308	241.95	255.01	0.0028	0.98	1.96	8.64	0.98
AC-2	298	153.31	194.18	0.0045	0.98	2.42	9.42	0.95
	303	158.25	196.02	0.0051	0.97	2.55	11.37	0.94
	308	161.21	194.09	0.0061	0.98	2.75	14.12	0.95
AC-2_MW_	298	164.45	200.01	0.0053	0.98	2.57	11.95	0.95
	303	169.21	195.82	0.0082	0.97	3.05	19.02	0.94
	308	175.70	194.18	0.0139	0.97	3.64	28.84	0.94

## Data Availability

Not applicable.

## Supplementary Materials

The following supporting information can be downloaded at: https://www.mdpi.com/article/10.3390/molecules28134922/s1, Figure S1: Pseudo-first order fitting model for the samples: (a) AC-1-OX_MW_, (b) AC-2, (c) AC-2_MW_; Figure S2: Pseudo-second order fitting model for the samples: (a) AC-1-OX_MW_, (b) AC-2, (c) AC-2_MW_; Figure S3: Intra-particle diffusion model for the samples: (a) AC-1-OX_MW_, (b) AC-2, (c) AC-2_MW_; Figure S4: Experimental adsorption isotherms of MB on tested carbon materials at: (a) 289 K, (b) 303 K and (c) 308 K; Figure S5: Linear fitting of adsorption isotherms according to Langmuir (a–f) and Freundlich (g–l) models.
