# Supplementary material for: Assessment of the Porous Structure and Surface Chemistry of Activated Biocarbons Used for Methylene Blue Adsorption
Source: Molecules. 2023 Jun 22;28(13):4922. doi: 10.3390/molecules28134922 (PMC10343508; doi:10.3390/molecules28134922)
Supplement: Supplementary file 1 [file molecules-28-04922-s001.zip › molecules-2462548-supplementary.pdf]

Supplementary Information

Assessment of the porous structure and surface chemistry of activated biocarbons used for Methylene Blue adsorption

Barbara Charmas [1,*], Magdalena Zięzio [1], and Katarzyna Jedynak [2]

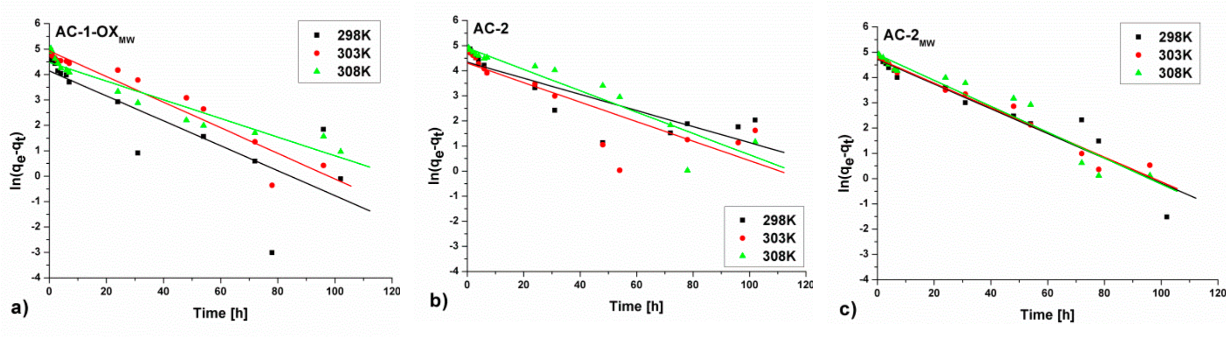

Figure S1. Pseudo-first order fitting model for the samples: (**a**) AC-1-OX$_{MW}$, (**b**) AC-2, (**c**) AC-2$_{MW}$.

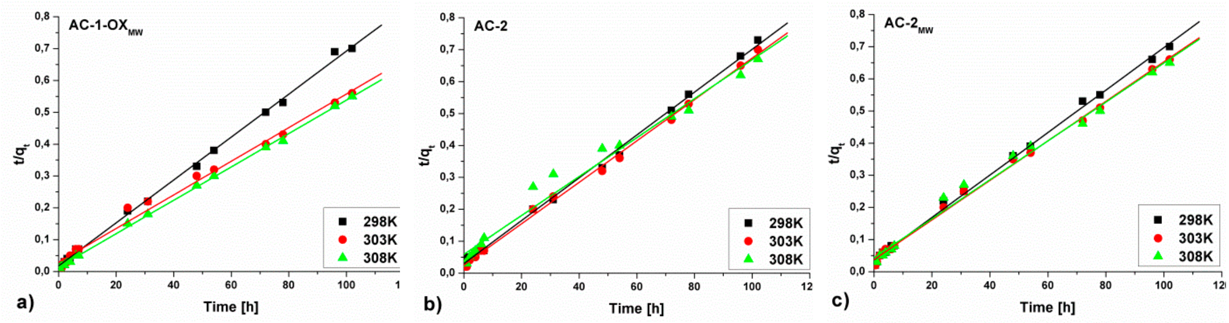

Figure S2. Pseudo-second order fitting model for the samples: (**a**) AC-1-OX$_{MW}$, (**b**) AC-2, (**c**) AC-2$_{MW}$.

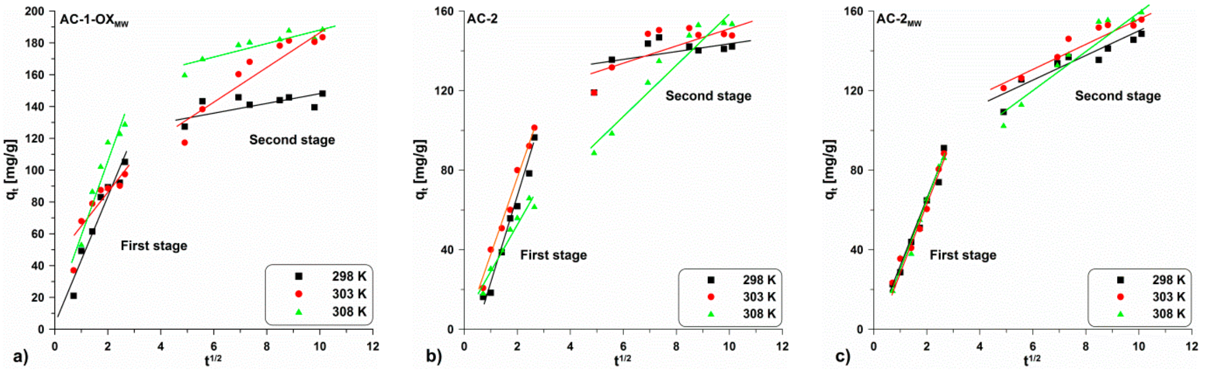

Figure S3. Intra-particle diffusion model for the samples: (**a**) AC-1-OX$_{MW}$, (**b**) AC-2, (**c**) AC-2$_{MW}$.

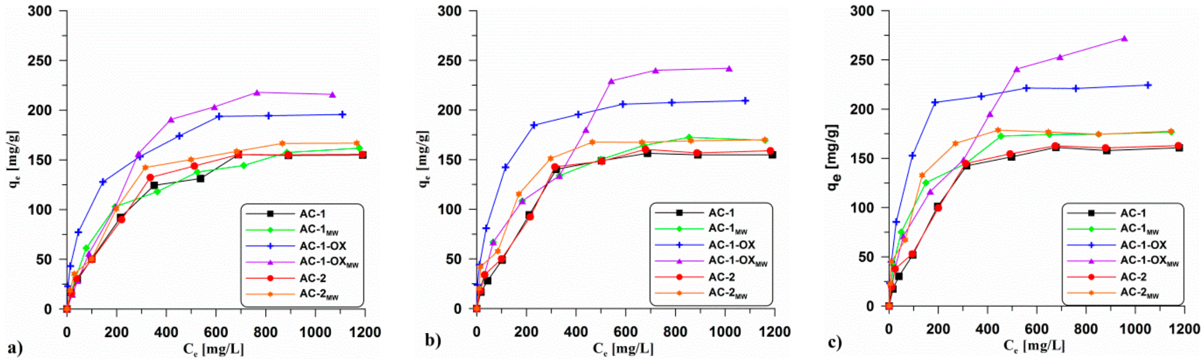

Figure S4. Experimental adsorption isotherms of MB on tested carbon materials at: (**a**) 289 K, (**b**) 303 K and (**c**) 308 K.

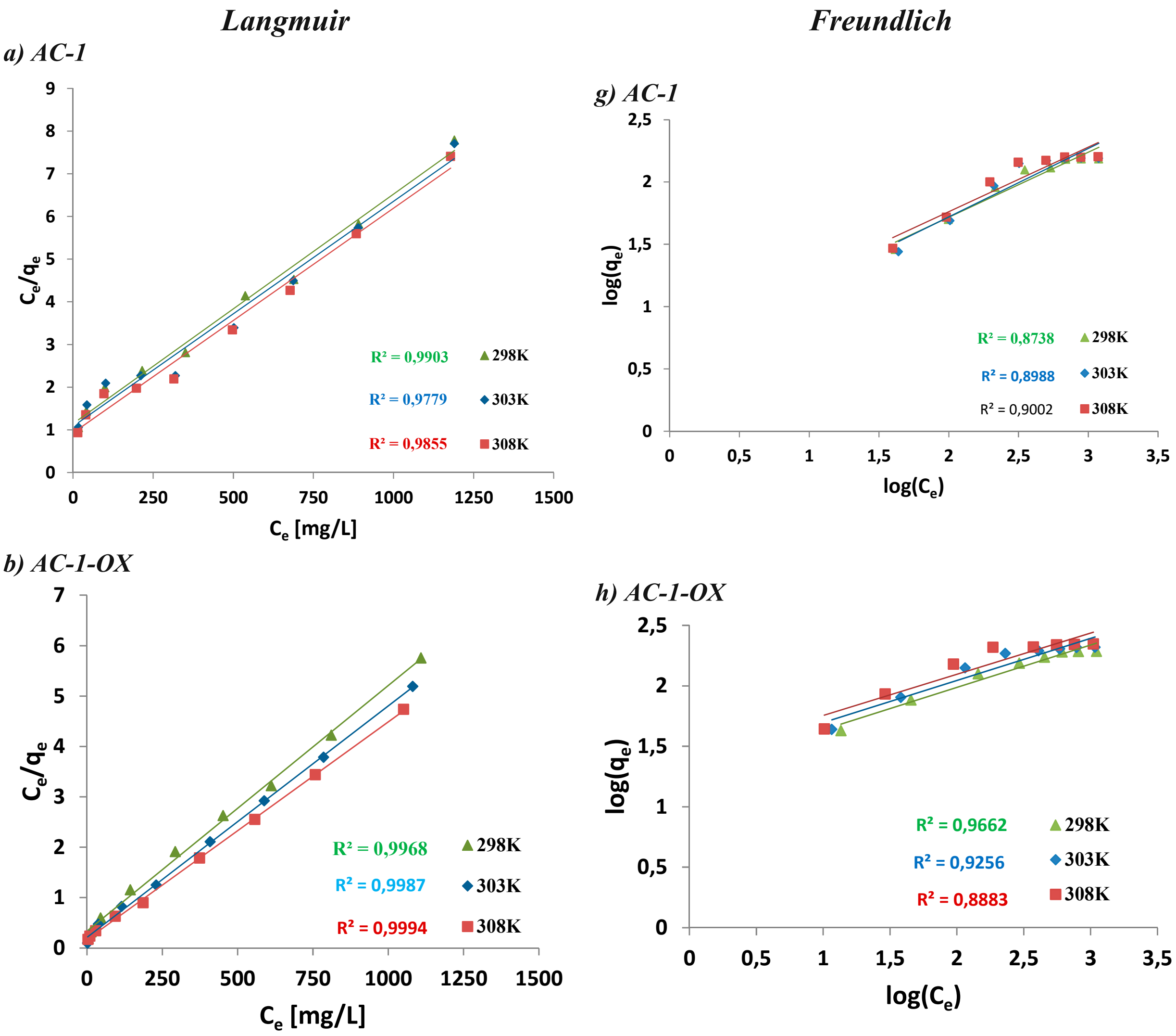

c) AC-1MW

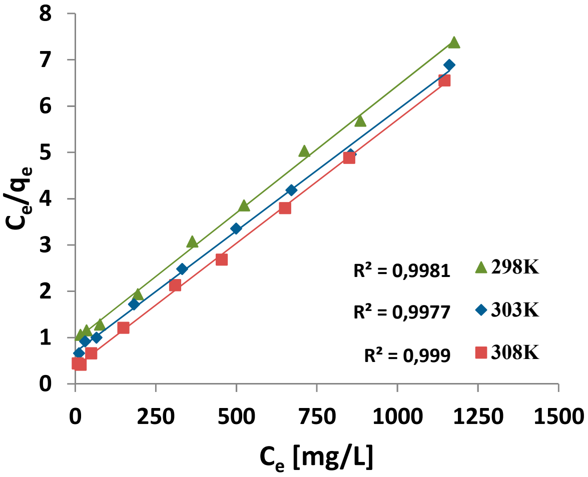

i) AC-1MW

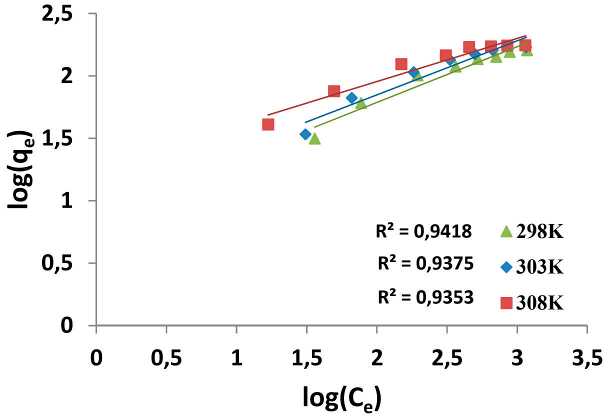

d) AC-1-OXMW

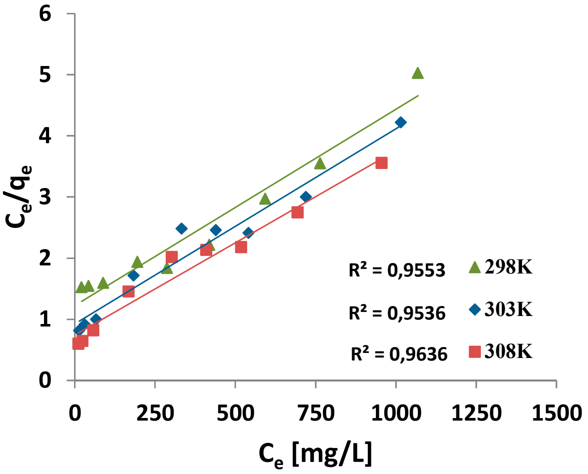

j) AC-1-OXMW

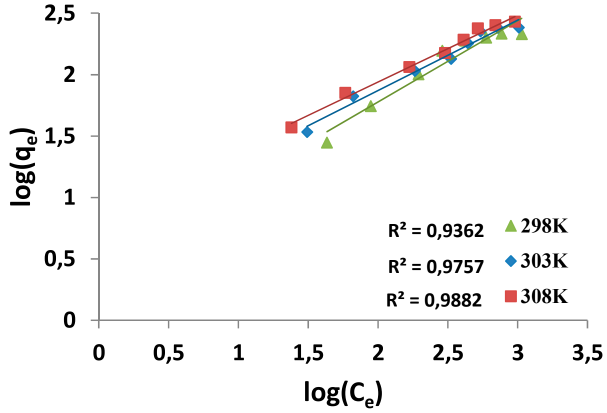

e) AC-2

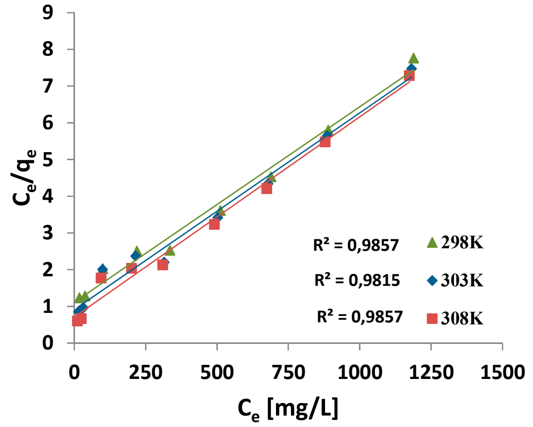

k) AC-2

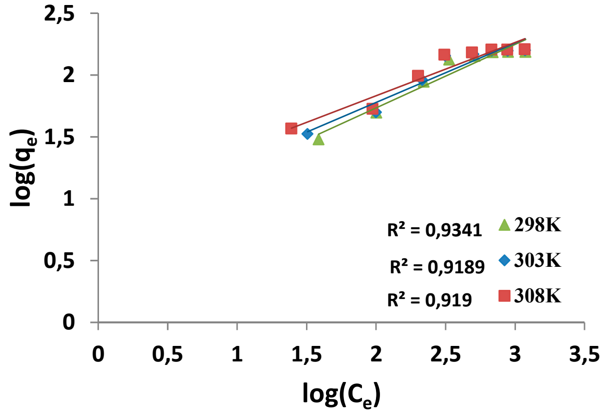

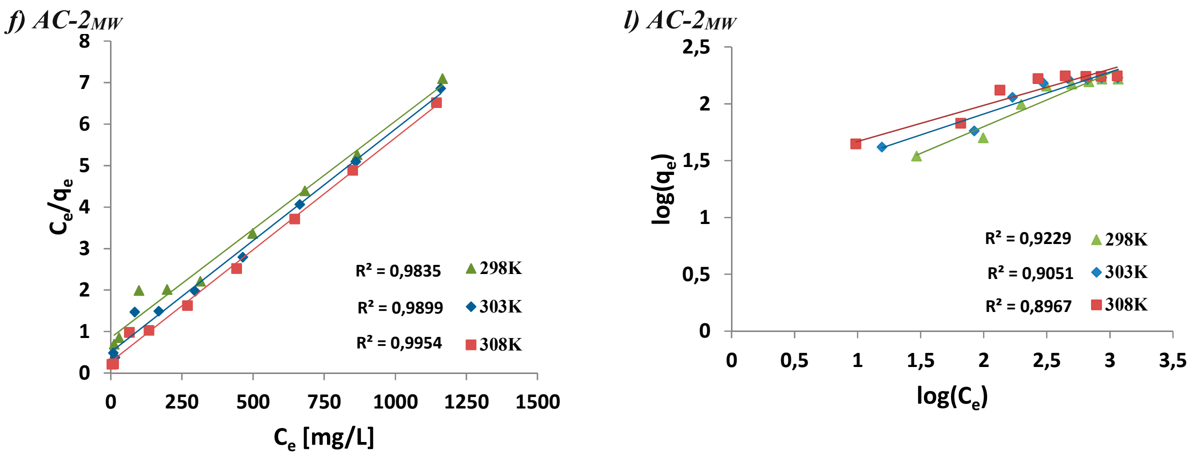

Figure S5. Linear fitting of adsorption isotherms according to Langmuir (a-f) and Freundlich (g-l) models.
